# Neurocognition and NMDAR co-agonists pathways in individuals with treatment resistant first-episode psychosis: a 3-year follow-up longitudinal study

**DOI:** 10.1038/s41380-024-02631-4

**Published:** 2024-06-07

**Authors:** Sara Camporesi, Lijing Xin, Philippe Golay, Chin Bin Eap, Martine Cleusix, Michel Cuenod, Margot Fournier, Kenji Hashimoto, Raoul Jenni, Julie Ramain, Romeo Restellini, Alessandra Solida, Philippe Conus, Kim Q. Do, Ines Khadimallah

**Affiliations:** 1 Center for Psychiatric Neuroscience, Department of Psychiatry, Lausanne University Hospital, Lausanne, Switzerland; 2 Service of General Psychiatry, Department of Psychiatry, Lausanne University Hospital, Lausanne, Switzerland; 3 Department of psychiatry and Emergency Department, Geneva University Hospital, Geneva, Switzerland; 4 Center for Biomedical Imaging, Ecole Polytechnique Fédérale de Lausanne (EPFL), Lausanne, Switzerland; 5 School of Pharmaceutical Sciences, University of Geneva, University of Lausanne, Geneva, Switzerland; 6 Center for Research and Innovation in Clinical Pharmaceutical Sciences, University of Lausanne, Lausanne, Switzerland; 7 Institute of Pharmaceutical Sciences of Western Switzerland, University of Geneva, University of Lausanne, Geneva, Switzerland; 8 Division of Clinical Neuroscience, Chiba University Center for Forensic Mental Health, Chiba, Japan; 9 Training and Research Institute in Mental Health (IFRSM), Neuchâtel Centre of Psychiatry, Neuchâtel, Switzerland; 10 Emergency medicine department, Geneva University Hospital, Geneva, Switzerland; 11 Psychiatry Department for Adults 2, Neuchâtel Centre of Psychiatry, Prefargier, Switzerland; 12 Translational Research Center, University Hospital of Psychiatry, University of Bern, Bern, Switzerland

**Keywords:** Diagnostic markers, Prognostic markers

## Abstract

This study aims to determine whether 1) individuals with treatment-resistant schizophrenia display early cognitive impairment compared to treatment-responders and healthy controls and 2) N-methyl-D-aspartate-receptor hypofunction is an underlying mechanism of cognitive deficits in treatment-resistance. In this case‒control 3-year-follow-up longitudinal study, n = 697 patients with first-episode psychosis, aged 18 to 35, were screened for Treatment Response and Resistance in Psychosis criteria through an algorithm that assigns patients to responder, limited-response or treatment-resistant category (respectively resistant to 0, 1 or 2 antipsychotics). Assessments at baseline: MATRICS Consensus Cognitive Battery; N-methyl-D-aspartate-receptor co-agonists biomarkers in brain by MRS (prefrontal glutamate levels) and plasma (D-serine and glutamate pathways key markers). Patients were compared to age- and sex-matched healthy controls (n = 114). Results: patient mean age 23, 27% female. Treatment-resistant (n = 51) showed lower scores than responders (n = 183) in processing speed, attention/vigilance, working memory, verbal learning and visual learning. Limited responders (n = 59) displayed an intermediary phenotype. Treatment-resistant and limited responders were merged in one group for the subsequent D-serine and glutamate pathway analyses. This group showed D-serine pathway dysregulation, with lower levels of the enzymes serine racemase and serine-hydroxymethyltransferase 1, and higher levels of the glutamate-cysteine transporter 3 than in responders. Better cognition was associated with higher D-serine and lower glutamate-cysteine transporter 3 levels only in responders; this association was disrupted in the treatment resistant group. Treatment resistant patients and limited responders displayed early cognitive and persistent functioning impairment. The dysregulation of NMDAR co-agonist pathways provides underlying molecular mechanisms for cognitive deficits in treatment-resistant first-episode psychosis. If replicated, our findings would open ways to mechanistic biomarkers guiding response-based patient stratification and targeting cognitive improvement in clinical trials.

## Introduction

Patients with treatment-resistant schizophrenia (TRS), accounting for 15 to 30% of schizophrenia cases [1], suffer from continuous symptoms, with higher disability rates than those who respond to first-line antipsychotic treatment [2]. To offset inconsistency in the definition of treatment-resistance, which prevents between-study comparison, Treatment Response and Resistance in Psychosis (TRRIP) consensus guidelines defined resistance as inadequate response to at least two trials of different first-line antipsychotics for a duration of at least 6 weeks each [3]. Although clinical evidence showed that TRS patients display poor cognition in the advanced phases of the disease, the neurocognitive profile of TRS individuals in the early phases of psychosis [4] is lacking. Moreover, underlying mechanisms of resistance are unclear. While most TRS subjects may benefit from early treatment with clozapine [5, 6], in clinical practice the diagnosis of treatment resistance can be delayed for many years, thus hindering the introduction of clozapine at an early stage [7], when the course of illness is still modifiable [6, 8]. It has been thus proposed that clozapine be prescribed in patients with limited-response schizophrenia (LRS), i.e., after only one failed treatment episode [9]. However, little is known about LRS.

Based on emerging evidence suggesting the involvement of glutamatergic transmission in TRS and LRS [10–13], we aim to characterize the neurocognitive profile and its underlying mechanisms in these individuals in the early phases of psychosis.

Given the resistance to D2 antagonist antipsychotics, TRS core pathophysiological features may implicate N-methyl-D-aspartate-receptor (NMDAR) hypofunction. As a Ca^2+^ permeable, ligand-gated cation channel, NMDAR plays a critical role in cognitive functions including memory, learning and synaptic plasticity. Its activation necessitates depolarization and the simultaneous binding of an agonist (glutamate) and co-agonist (D-serine or glycine, with the former being considered as the main ligand in the adult brain) to their respective binding sites. Glycine is synthetized from L-serine through the enzyme serine hydroxymethyltransferase 1 (SHMT1). Virtually all brain D-serine is synthetized de novo from L-serine through the enzyme serine racemase (SRR). Subsequently, D-serine undergoes degradation through D-amino-acid oxidase (DAAO). NMDAR hypofunction mediated by dysregulation of the pathways involving its co-agonists (Figs. 3A, 4A) has been documented in patients with psychosis [14, 15]. Indeed, increases in prefrontal cortex (PFC) glutamate levels, reported in small patient cohorts resistant to first-line treatment [10–13], were recently confirmed in a larger study of first-episode patients (FEP) [16]. The neuronal excitatory amino acid transporter 3 (EAAT3) for glutamate and cysteine has also been linked to glutamatergic neurotransmission and redox disruption in schizophrenia [17–21]. Moreover, evidence of abnormalities in the D-serine pathway highlights its critical role in schizophrenia pathogenesis: these include risk gene encoding the enzymes SRR and DAAO, altered levels of D-serine, L-serine, glycine as well as SHMT1 [15, 22–29]. Additionally, the SRR-knockout mouse model recapitulates reduction in NMDAR function and most of the phenotypic characteristics of schizophrenia, including structural pathology, EEG abnormalities and impaired cognition [30]. Only two studies assessed D-serine pathway markers in TRS [31, 32], albeit with conflicting results: indeed, both were based on rather small cohorts with chronic schizophrenia and compared patients with heterogeneously defined treatment resistance to a group of healthy controls (HC) but not to responders (RESP).

Clozapine, beneficial to most TRS patients [5, 6], shows low affinity for D2-receptors and appears to modulate NMDAR activity [33, 34]. Indeed, there is preclinical evidence of clozapine modulating NMDAR function [35]: clozapine could rescue D-serine and glutamate levels in induced-pluripotent-stem-cell (iPSC)-derived astrocytes from patients with clozapine-responsive schizophrenia [36], and could restore electrophysiological properties and glutamatergic signaling in iPSC-derived glutamatergic neurons [35, 37] from patients with schizophrenia; clozapine, unlike other antipsychotics, has been demonstrated to inhibit the transport of synaptosomal glycine in vitro, potentially enhancing NMDAR function [38]. Moreover, on the clinical level, clozapine administration in TRS patients led to increases in plasma D-serine/L-serine ratio [32]. Clozapine-mediated glutamatergic biomarkers rescue could also explain the absence of improvement in clinical trials investigating antipsychotic augmentation therapy with NMDAR co-agonists in patients already taking clozapine [39, 40], further suggesting that the clozapine response in TRS might be mediated by glutamatergic modulation.

In this study, we first determine whether the TRS group would show cognitive deficits compared to the RESP group in a prospective cohort of FEP. We characterize the newly defined LRS group, determining whether it shares the early cognitive impairment of TRS compared to RESP. To reveal the underlying mechanisms of treatment resistance, we compare markers of glutamatergic dysregulation between all groups by assessing NMDAR co-agonist pathways in plasma and brain. Finally, we investigate the relationship between these markers and cognition.

## Methods

### Study population

Patients were recruited between 2005 and 2021 from the prospective ongoing TIPP (Treatment and early Intervention in Psychosis Patients) Lausanne cohort [41]. TIPP program inclusion criteria are the following: (i) age between 18 and 35 years, (ii) having a first episode psychotic disorder, defined as reaching the “psychosis threshold” subscale on the Comprehensive Assessment of At Risk Mental States scale (CAARMS) and (iii) antipsychotic treatment was initiated no more than 6 months before inclusion, regardless of the number of molecules and dosage that the patient had received.

**Fig. 1:**
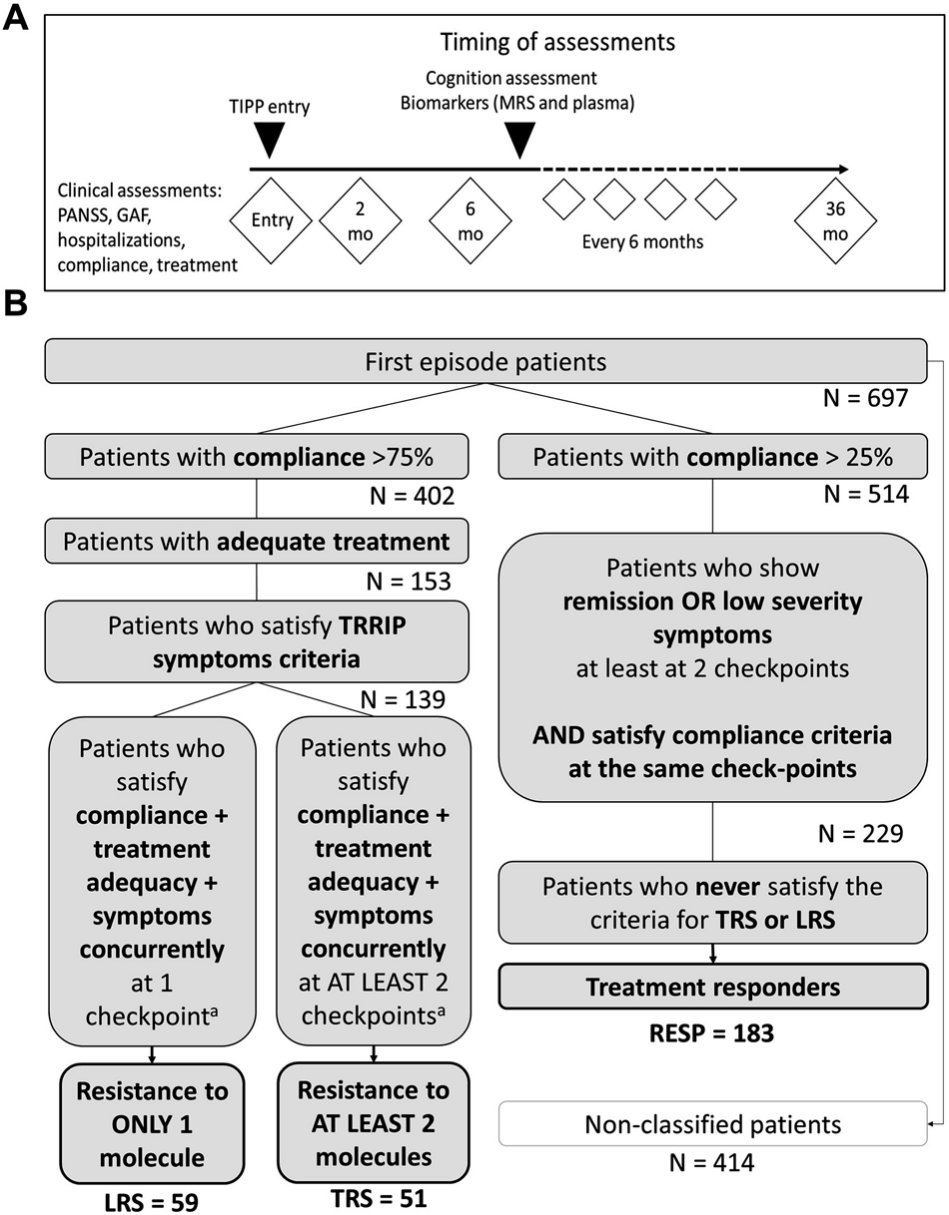
Timeline of assessments and patient stratification. **A** Clinical data (PANSS, GAF, hospitalizations, treatment compliance, antipsychotic molecule and dosage) was collected prospectively at TIPP entry and at months 2, 6, 12, 18, 24, 30 and 36. Moreover, cognitive tests, blood tests and MRS were performed as soon as patients participating in the TIPP program were willing and sufficiently stable to undergo these assessments. The mean delay between TIPP entry and the abovementioned assessments was 10 months. **B** The TRRIP algorithm was specifically coded to allow for patient stratification based on the TRRIP criteria. Clinical assessment of TRS, LRS and RESP patients over a 3-year longitudinal follow up was obtained using standardized rating scales (PANSS and GAF) at 2, 6, 12, 18, 24, 30 and 36 months. After selecting patients for whom we had data on compliance (ascertained both clinically, with patients taking >75% of prescribed treatment, and by blood antipsychotic level), and on treatment dosage (at least 6 weeks at mid-point of licensed therapeutic dosage), we selected patients who showed: (i) at least two moderate PANSS symptom-scores ( ≥ 4) or at least one severe score ( ≥ 5); (ii) at least moderate functional impairment ( < 61 GAF score); when scores were lacking, but the patient still met the compliance and dosage criteria for a given timepoint, we used prospective data on hospitalization as proxy: if the patient was hospitalized, with psychotic symptoms being the main reason for hospitalization, this was considered a severe PANSS score. This process was repeated for each timepoint. In case of persistence of psychotic symptoms despite adequate antipsychotic treatment dosage and duration, the number of failed treatment episodes was used to define LRS (i.e., resistance to one drug) and TRS (i.e., resistance to at least 2 drugs). To identify RESP patients, the following criteria were used: in the presence of treatment compliance (the threshold considered for RESP was >25% of prescribed treatment), patients were labeled as responders (i) if they met the Andreasen criteria [85] for remission for at least 6 months or (ii) if impairment on a standardized scale (PANSS or GAF) was rated as mild or lower at at least two checkpoints. Finally, patients who met the preceding criteria but satisfied the resistance symptoms criteria for at least one checkpoint were excluded. We identified 51 TRS patients, 59 LRS patients and 183 RESP patients. 414 patients were not classified due to incomplete data on compliance.

All patients (N = 697) underwent prospective clinical assessment over the 3-year program: clinical (Positive and Negative Symptoms Scale [PANSS] and Global Assessment of Functioning [GAF]) and medication status (molecule, dosage and compliance) were assessed at TIPP entry, at 2 months, and prospectively every 6 months (Fig. 1A). A subgroup participated in neurobiological research (N = 251) and underwent, among other analyses, blood tests and magnetic resonance spectroscopy (MRS) assessment as soon as the patients were willing and clinically able to undergo these tests. This study was carried out in accordance with the Declaration of Helsinki and was approved by the local Ethics Committee (Commission cantonale d’éthique de la recherche sur l’être humain (CER-VD)). All patients gave their informed consent.

FEP were compared to a group of gender-and-age-matched HC (N = 114) recruited following the Diagnostic Interview for Genetic Studies (DIGS) [42] criteria, to confirm the absence of any major psychiatric or substance use disorders; control individuals were excluded if they had a history of severe head trauma, if they were taking any medication or if they presented with acute illness at the time of undergoing biomarker and cognitive assessment; furthermore, having a first-degree relative with psychotic disorder constituted an exclusion criteria.

### Patient assignment to the TRS, LRS and RESP categories

Patients were stratified according to treatment response and resistance through the “TRRIP algorithm” (detailed in Fig. 1B), an SPSS-coded algorithm designed to implement the TRRIP criteria [3] based on prospective clinical data and standardized rating scales: Positive and Negative Symptoms Scale (PANSS) [16] and Global Assessment of Functioning (GAF) [43]. The algorithm was implemented considering data on compliance, treatment and symptoms for each one of the 7 prospective timepoints. For each timepoint, after selecting patients for whom we had data on compliance, ascertained both clinically (at least 75% of pills taken) and by blood antipsychotic level, and on treatment dosage (at least 6 weeks at mid-point of licensed therapeutic dosage), we selected patients who showed: (i) at least two moderate PANSS symptoms-scores ( ≥ 4) or at least one severe score ( ≥ 5); (ii) at least moderate functional impairment ( < 61 GAF score) at that given timepoint; when scores were lacking, but the patient still met the compliance and dosage criteria, we used prospective data on hospitalization as proxy: if the patient was hospitalized, with psychotic symptoms being the main reason for hospitalization, this was considered a severe PANSS score for the corresponding timepoint. This process was repeated for each timepoint. In case of persistence of psychotic symptoms despite adequate antipsychotic treatment dosage and duration, the number of failed treatment episodes was used to define LRS (i.e., resistance to one drug) and TRS (i.e., resistance to at least 2 drugs). RESP were defined as patients who still satisfied the compliance (at least 25% of pills taken) and treatment threshold criteria but showed either persistent remission, i.e., response for more than 6 months, or symptoms that did not cross the severity thresholds of TRS criteria after one line of appropriate antipsychotic therapy.

#### Determination of compliance by antipsychotic plasma concentrations

To ascertain compliance for LRS ant TRS patients, antipsychotic plasma concentrations were assessed at least once in conjunction with a failed treatment episode. If a patient was considered as compliant on a clinical level, but plasma antipsychotic concentrations indicated a lack of treatment compliance, the patient was considered non-compliant.

Blood samples were obtained in the morning in fasting conditions to assess antipsychotic plasma levels. Liquid chromatography/mass spectrometry methods were used for measuring olanzapine, risperidone, OH-risperidone, quetiapine, amisulpride, and aripiprazole plasma levels as previously described [44, 45].

All methods are used on a routine basis in our accredited laboratory (ISO 15189 and 17025), with external quality controls (LGC Standards Proficiency Testing [Teddington, United Kingdom]; Arvecon [Walldorf, Germany]; Quality Control Centre Switzerland [Chêne-Bourg, Switzerland]). Patients were considered compliant when antipsychotic plasma levels were in the recommended therapeutic range [46]. For risperidone, the sum of risperidone and of its metabolite 9-OH risperidone was used.

Patients receiving long-acting injectable antipsychotic drugs at equal or higher doses than mid-point of licensed therapeutic dosage and who satisfied the other treatment-resistance criteria were assigned to the LRS or TRS category even when biological assessment of compliance was not available.

### Neurocognition profile, brain neurochemical and plasma biochemical assessments

MRS, blood tests and cognitive assessment were performed at the same timepoint for each patient, usually over two days. The mean delay between entry in the TIPP program and participation in the above-mentioned assessments was 10 months (Fig. 1A). This delay was due to the fact that minimal clinical stabilization was needed for the patient to undergo the tests and implied that most patients were not drug naïve at the time of cognitive, neurobiological and neuroimaging assessment.

Neurocognitive assessment was performed through the MATRICS Consensus Cognitive Battery [47–49].

Levels of glutamate, glutamine and glycine in the medial prefrontal cortex were assessed by MRS as previously described [50], using a 3 Tesla magnetic resonance scanner (Magnetom TimTrio, Siemens Healthcare) with a transverse electromagnetic (TEM 3000) head coil (MR Instruments, Inc). We adjusted first- and second-order shims using FAST(EST)MAP [51] to optimize the homogeneity of the magnetic field. We acquired in vivo 1 H MR spectra from a volume of interest (20×20×25 mm3) located in the medial prefrontal cortex; this was obtained using a full-intensity short-TE spin-echo localized single-voxel spectroscopy technique (SPECIAL) [52], after applying outer volume suppression and water suppression with variable-pulse power and optimized relaxation delays. The subsequent parameters were used: acquisition bandwidth was 2 kHz, echo time/repetition time was 6/4000 ms, number of averages was 148, vector size was 2048. Moreover, to estimate the tissue composition in the volume of interest, 3D T1-weighted images were acquired through the use of a magnetization-prepared rapid gradientecho (MPRAGE) sequence with a 32-channel head array coil; the following parameters were used: echo time/repetition time was 2.98/2300 ms, inversion time was 900 ms, flip angle was 9°, slice thickness was 1.2 mm, matrix was 240 × 256, field of view was 240 × 256 mm^2^.

Plasma biochemical measurements of D-serine and glutamate pathways amino acids glutamate, glutamine, glycine and L-serine (Figs. 3A,4A) were measured according to standardized procedures using a column-switching high-performance-liquid-chromatography (HPLC) system (Shimadzu Corporation, Kyoto, Japan) combined with fluorescence detection as previously described [22]; to minimize the effect of amino acids originating from food sources, fasting blood samples were obtained ( > 3 h without any meals and/or nutritious drinks). Manufacturers procedures were followed to assess plasma protein levels of SRR: E15214h Human SRR/Serine racemase ELISA Kit (cat Number: E15214h) from EIAaB Supplier (researchd.com), SHMT1 (abx383190 Human SHMT1 ELISA Kit | Abbexa Ltd) and EAAT3 (E-EL-H5591 elabscience.com). The immuno-assay’s validity has been confirmed using recombinant proteins for each target molecule, with known concentrations. A curve was constructed to illustrate the correlation between the target molecule’s concentration and the signal intensity detected by the kit. Kit precision evaluation included verifying whether samples of established concentrations align with the standard curve. Additionally, background noise (BLCN) was taken into consideration to ensure precise and dependable measurements in the assessment process.

### Statistical analysis

The statistical analyses were conducted using IBM SPSS Statistics 28.0.1 and JMP software (JMP® Pro 17.0.0; 622753). To ensure the suitability of the variables included in the preliminary analysis, the Shapiro-Wilk test for normality was performed, and all variables were conformed with the normal distribution hypothesis. Furthermore, no significant interactions between the variables of interest and age or sex were observed, indicating that the effects of these demographic factors did not alter the relationship among the variables. For group comparisons, a one-way ANOVA was initially conducted, followed by Student’s t-test. A significance threshold of α = 0.05 was used for all statistical tests. To address the issue of multiple comparisons and control the overall rate of false positives (type I error), we used the False Discovery Rate adjustment method (FDR) which effectively manages the increased likelihood of observing false positive results when running multiple statistical tests. Furthermore, to provide valuable information for interpreting obtained results, a 95% confidence interval was calculated to estimate the range of values, with lower and upper levels.

## Results

### Patients’ stratification

Implementing the TRIPP algorithm, out of 697 FEP, 293 individuals were classified as TRS (n = 51), LRS (n = 59) or RESP (n = 183) (Fig. 1B). 414 patients were not assigned to any category largely due to the fact that data on treatment molecules and compliance started to be collected in June 2010 (Supplementary Table 1). The TRS and RESP groups showed no difference concerning sex, ethnicity or age at psychosis onset; at baseline, i.e. at the time of biomarker and cognitive assessment, there was no difference between groups in age, duration of untreated psychosis, body mass index, or diagnosis. The age at psychosis onset was slightly higher in LRS than in RESP individuals. The mean chlorpromazine equivalent at baseline was higher in TRS (mean ± STD; 565 ± 323 mg/day) than in RESP (285 ± 199 mg/day;p < 0.001) (Table 1). No patient was taking clozapine at baseline. At the time of biomarker and cognitive assessment, 134 patients out of the 293 classified patients were already deemed to have achieved a full response to treatment. Notably, none of the TRS patients exhibited an initial response to any antipsychotic.

**Table 1:** Demographic table.

Variable	HC^a^ (114)	Patients			RESP vs TRS		RESP vs LRS	
		RESP^b^ (183)	TRS^c^ (51)	LRS^d^ (59)	*P*-value	Cohen’s d (df^e^)	*P*-value	Cohen’s d (df)
Age at psychosis onset, mean (SD^f^), y^g^		23.4 (5.0)	22.1 (5.2)	24.0 (4.8)	0.097	0.268 (222)	**0.0451**	−0.117 (227)
Age baseline, mean (SD), y	25.4 (4.9)	24.8 (5.0)	23.5 (5.2)	25.9 (4.9)	0.219	0.245 (170)	0.214	−0.235 (174)
CPZ^h^ equivalent, mean (SD), mg/day	–	285 (199)	565 (323)	384 (214)	**<0.001**	−1.162 (66)	0.072	−0.487 (66)
Body mass index, mean (SD)	22.8 (2.8)	24.5 (3.7)	24.6 (4.9)	23.9 (4.7)	0.898	−0.037 (59)	0.599	0.148 (60)
Disorder duration^i^, mean (SD), days	–	106 (246)	116 (246)	166 (333)	0.883	−0.039 (66)	0.436	−0.217 (64)
Gender								
Male, no^j^ (%)	73 (64%)	130 (71%)	39 (76%)	44 (75%)	0.47	χ2 (0.56)	0.40	χ2 (0.56)
Female, no (%)	41 (36%)	53 (29%)	12 (24%)	15 (25%)				
Ethnicity^k^, no.	94 C, 5 A, 6 M, 9 O	55 C, 8 A, 3 M, 12 O	13 C, 3 A, 1 M, 8 O	10 C, 6 A, 4 M, 10 O	NA	NA	NA	NA
Diagnosis no.					NA	NA	NA	NA
Schizophrenia	–	100	37	46				
Schizophreniform disorder	–	22	3	3				
Schizoaffective disorder	–	16	6	7				
Depression w/ psychotic feat.	–	10	2	1				
Bipolar disorder	–	14	0	0				
Other^l^	–	21	3	2				
Years of education, mean (SD)								
Patient	15.6 (2.4)	12.9 (2.7)	11.2 (2.6)	12.1 (2.5)	**0.022**	0.643 (62)	0.287	0.294 (62)
GYOE^m^ patient	2.51 (0.52)	1.96 (0.67)	1.63 (0.60)	1.79 (0.63)	0.074	0.497 (62)	0.362	0.251 (62)
Mother	13.0 (3.5)	11.9 (4.4)	11.1 (4.4)	9.17 (4.0)	0.589	0.170 (46)	0.067	0.635 (43)
GYOE mother	2.04 (0.66)	1.91 (0.72)	1.73 (0.70)	1.33 (0.65)	0.435	0.245 (46)	**0.020**	0.816 (43)
Father	13.9 (4.3)	12.9 (5.0)	12.5 (5.0)	9.3 (5.1)	0.809	0.080 (43)	**0.039**	0.720 (42)
GYOE father	2.16 (0.69)	2.00 (0.84)	2.00 (0.71)	1.42 (0.79)	1.000	0.000 (43)	**0.044**	0.703 (42)

The TRS and RESP group showed no difference concerning gender, age at psychosis onset, age at baseline, duration of untreated psychosis, BMI, ethnicity, or diagnosis. The age at psychosis onset was slightly higher in LRS patients than in RESP. The mean chlorpromazine equivalent was higher in TRS patients than in RESP. RESP displayed a higher number of years of education than TRS patients; mothers and fathers of RESP patients showed higher grades of attained education than LRS patients.

Statistically significant *p*-values are in bold.

^a^Healthy controls.

^b^Responders.

^c^Patients with treatment resistant schizophrenia.

^d^Patients with limited response schizophrenia.

^e^Degree of freedom.

^f^Standard deviation.

^g^Years.

^h^Chlorpromazine.

^i^Before entering the TIPP program.

^j^Number.

^k^Ethnicity: C, Caucasian; A, African; M, mixed; O, other.

^l^Other includes schizotypal personality disorder, autism spectrum disorder, brief psychotic disorder, psychotic disorder not otherwise specified.

^m^Grade of attained education.

### Clinical profile: social functioning and hospitalization rate

**Fig. 2:**
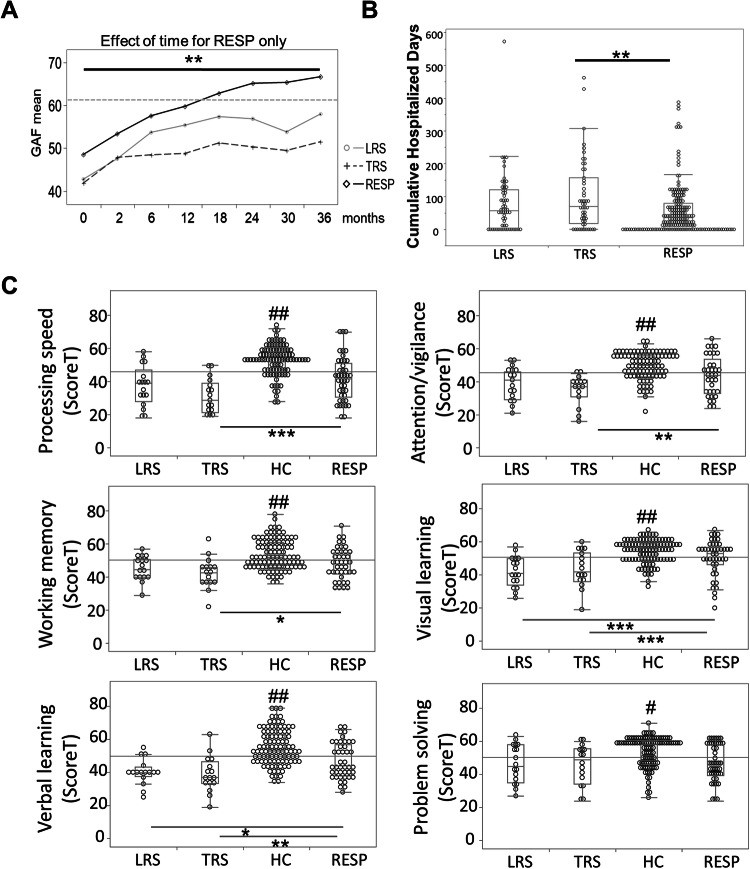
Clinical and cognitive profile for TRS, LRS, RESP and HC groups. **A** Evolution of social functioning over 36 months and cumulative days of hospitalization by group: already in the early months of follow-up TRS and LRS show significantly lower functioning at each time point compared to RESP and never reach the threshold of 61. An effect of time is observed in RESP but not in LRS or TRS. **B** The discrimination between the TRS and RESP groups was also highlighted by the number of cumulative days of hospitalization over a 3-year period. **C** Neurocognitive profile at baseline in HC, RESP, LRS and TRS: HC display higher scores in all cognitive domains compared to all patient groups (RESP, LRS and TRS; p ≤ 0.01 for HC versus each group #; p ≤ 0.001 for HC versus each group ##). TRS patients displayed lower T scores than RESP in 5 out of 6 domains (respective mean ± std dev.): processing speed (30 ± 12, 41 ± 13; p = 0.001), attention/vigilance (35 ± 9, 43 ± 12; p = 0.005), visual learning (43 ± 11, 50 ± 11; p = 0.001), working memory (42 ± 9, 48 ± 9; p = 0.030) and verbal learning (38 ± 11, 46 ± 11; p = 0.010). LRS patients scored lower than RESP only in verbal learning (40 ± 8 p = 0.04) and visual learning (42 ± 9, p = 0.001). P two-tailed; p ≤ 0.05 *, p ≤ 0.01 **, p ≤ 0.001 ***; FDR adjusted.

At baseline, TRS displayed lower GAF scores than RESP (mean difference 6.07 [95% CI, 1.10-11.04]). During the 3-year follow-up, compared to RESP, both LRS and TRS patients had poorer functioning and never reached the threshold of 61. This result was expected given the use of the GAF score as a selection criterion for TRS and LRS. However, a time effect over 3 years was present only in RESP, showing that poorer functioning is an early and continuous feature of TRS and of LRS (Fig. 2A). The discrimination between the TRS and RESP groups was also highlighted by the number of cumulative days of hospitalization (mean difference 43.2 days [95% CI, 16.5-69.9; p = 0.001) (Fig. 2B). Both TRS and LRS thus showed a persistent lower functioning over the 3-year follow-up, with LRS patients having an intermediary phenotype between TRS and RESP.

### Neurocognitive profile

We compared the neurocognitive profile between TRS, LRS, RESP and HC. TRS displayed lower T scores than RESP in five tasks (respective mean ± std dev.): processing speed (30 ± 12, 41 ± 13; p = 0.001), attention/vigilance (35 ± 9, 43 ± 12; p = 0.005), visual learning (43 ± 11, 50 ± 1; p = 0.001), verbal learning (38 ± 11, 46 ± 1; p = 0.010) and working memory (42 ± 9, 48 ± 9; p = 0.030). LRS scored lower than RESP only in verbal learning (40 ± 8; p = 0.039) and visual learning (42 ± 9; p = 0.001). Patient groups displayed lower scores in all domains than the HC (Fig. 2C). These results highlight that cognitive deficit was already present in the early phase of psychosis with worse cognitive profile in the TRS group compared to RESP, whereas LRS displayed an intermediate phenotype between RESP and TRS.

### NMDAR co-agonists pathway biomarkers

**Fig. 3:**
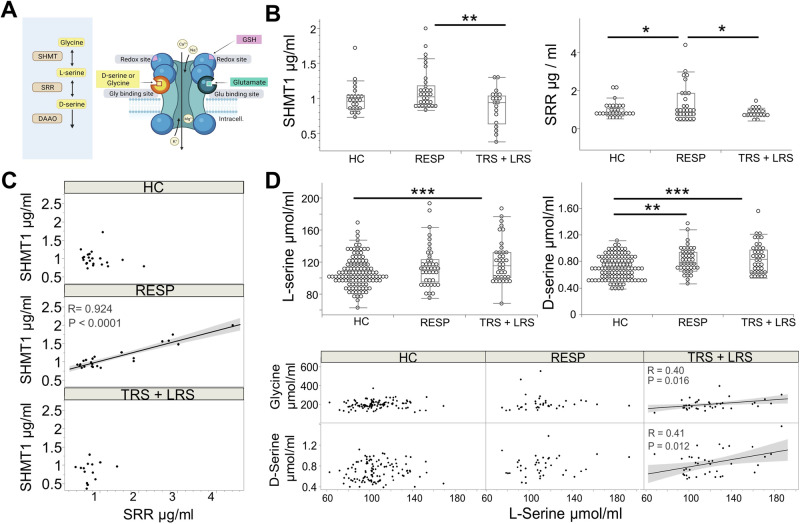
D-serine pathway. **A** Regulation of NMDAR activation: **left panel** shows the D-serine metabolic pathway: glycine is synthetized from L-serine via serine hydroxymethyltransferase 1 (SHMT1). Virtually all brain D-serine is derived from de novo synthesis from L-serine via serine racemase (SRR). In turn, D-serine is degraded through D-amino acid oxidase (DAAO). NMDAR is only activated when depolarization and both co-agonists (D-serine and glutamate) are present (**right panel**); reduced glutathione (GSH), through binding at the redox site, can potentiate NMDAR activity. **B** The treatment resistant group TRS + LRS displayed a dysregulation in D-Serine pathway, with SRR and SHMT1 activities as limiting factors: SHMT1 and SRR plasma levels were higher in RESP versus TRS + LRS. SRR plasma level was higher in RESP versus HC. **C** The positive correlation between SRR and SHMT1 in RESP was absent in TRS + LRS. **D** Compared to HC, plasma concentration of D-serine was higher in patients, and concentration of L-serine was higher in TRS + LRS (**higher panel**). The positive association between levels of D-serine and L-serine (reflecting SRR enzyme activity regulation) and of L-serine and glycine (reflecting SHMT1 regulation) in TRS + LRS but not in RESP, suggests that the enzymatic activity of SRR and SHMT1 could be key limiting factors in TRS + LRS (**lower panel**).

**Fig. 4:**
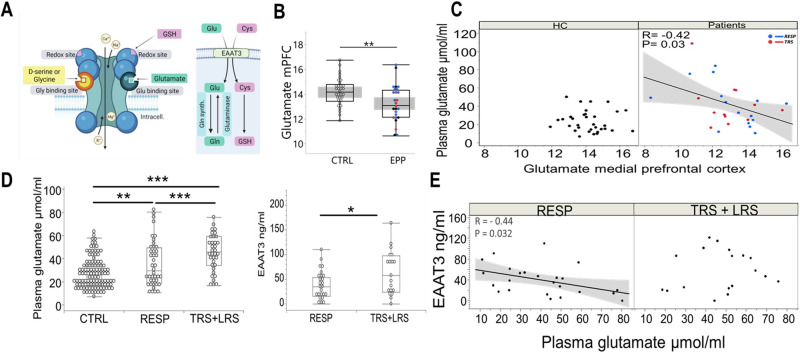
Glutamate pathway. **A** The glutamate metabolic pathway and the central role of EAAT3 in transport and regulation. The excitatory amino acid glutamate is synthesized by glutaminase and metabolized by glutamine synthetase. L-glutamate is metabolized to the inhibitory amino acid gamma-aminobutyric acid (GABA) by glutamic acid decarboxylase. The Excitatory Amino Acid Transporter 3 (EAAT3), located predominantly at the neuronal level, is a co-transporter of glutamate and cysteine, the latter being the key limiting substrate of glutathione (GSH). Hence, EAAT3 is critically involved in the synthesis of intracellular GSH and its ensuing protection from oxidative stress. Also, GSH can potentiate NMDAR activity by binding at its redox site (**left panel**). EAAT3 thus contributes to the subtle regulation of glutamatergic/NMDAR neurotransmission [57]. **B** Prefrontal cortex glutamate concentrations were higher in controls (mean 14,15 ± 1,10]) than in patients (mean 13,24 ± 1,46, p = 0.008). Red dots: TRS; black dots: LRS; blue dots: RESP. **C** Association of brain and plasma glutamate in patients and HC. In patients, low prefrontal cortex glutamate levels assessed by MRS were associated (R = -0.42, p = 0.03) with high plasma glutamate levels (blue dots: RESP; red dots: TRS + LRS). **D** Plasma glutamate levels were higher in TRS + LRS versus RESP and HC (**left panel**) and glutamate transporter EAAT3 plasma levels were higher in TRS + LRS subjects versus RESP (mean difference 25.36 [95%CI, 4.63-46.09], p = 0.020) (**right panel**). Altogether, these results suggest a dysregulation of glutamate transporters in TRS + LRS. **E** A negative correlation between EAAT3 and glutamate plasma levels (R = - 0.44, p = 0.030) observed in the RESP group was disrupted in TRS + LRS, suggesting that the glutamate transporter EAAT3, key limiting factor in RESP, is dysregulated in TRS.

To test whether NMDAR hypofunction could underlie the observed cognitive deficit, we assessed key markers of the D-serine (Fig. 3A) and glutamate pathways (Fig. 4A) in HC, RESP, and individuals with treatment resistance. TRS and LRS groups showed no difference concerning these markers (Supplementary Fig. 1): therefore, they were pooled in the same TRS + LRS group.

#### D-serine pathway

Characterizing the function of key metabolizing enzymes of D-serine pathway (Fig. 3A), SHMT1 plasma concentrations were higher in RESP than in TRS + LRS (mean difference 238.18 [95% CI, 89.27-387.11]; p = 0.002). SRR levels were higher in RESP than in TRS + LRS (mean difference 480.22 [95% CI,89.15-871.29]; p = 0.017) and in HC (Fig. 3B). Testing the regulation of these two sequential enzymes, the observed positive correlation between the L-serine synthesis SHMT1 and degradation SRR enzyme levels in RESP (r = 0.924; p < 0.001) were disrupted in TRS + LRS (Fig. 3C). Moreover, L-serine and D-serine were increased in both RESP and TRS + LRS (p < 0.001 for both racemates) compared to HC, with no difference in D-serine, L-serine or glycine in TRS + LRS compared to RESP (Fig. 3D, higher panel). These combined results suggest that the D-serine pathway was dysregulated in the TRS + LRS compared to RESP.

The appearance only in the TRS + LRS subgroup of a positive correlation between glycine and L-serine, the substrate and product of SHMT1 respectively (r = 0.39;p = 0.010), as well as between L-serine and D-serine, the substrate and product of SRR respectively (r = 0.41;p = 0.010) (Fig. 3D, lower panel), further suggests that SHMT1 and SRR enzyme activities may represent limiting factors in treatment resistant individuals.

#### Glutamate pathway

Prefrontal cortex glutamate concentrations were evaluated in both patients and healthy controls (Fig. 4B). The mean glutamate levels in patients were lower (mean 13.24 [SD 1.46], p = 0.08) compared to controls (mean 14.15 [SD 1.10]). Unfortunately, the very limited number of subjects in other subgroups rendered the study statistically underpowered to draw meaningful conclusions. Further characterizing the glutamate pathway (Fig. 4A) and testing whether blood glutamate could reflect their brain levels as assessed by MRS, we found that low brain glutamate was associated with high blood glutamate levels in patients (r = -0.42; p = 0.027) but not in HC (Fig. 4C). We thus compared their plasma levels between TRS + LRS and RESP subgroups: plasma glutamate concentrations were increased in patients, with a higher level in TRS + LRS compared to RESP (mean-difference 12.97 [95%CI,5.37-20.57]; p < 0.001) (Fig. 4D, left panel). These results pointed to a dysregulated transport system of glutamate. Among various glutamate transporters analyzed in skin-derived fibroblasts of individuals with long-term schizophrenia, preliminary results showed mRNA levels abnormalities of the glutamate and cysteine transporter EAAT3 (Fig. 4A; Supplementary Fig. 2). Focusing then on the latter, EAAT3 protein plasma levels were higher in TRS + LRS than in RESP (mean-difference 25.36 [95% CI,4.63-46.09]; p = 0.020) (Fig. 4D, right panel). Moreover, a negative correlation between EAAT3 and glutamate plasma levels, observed in the RESP group (r = -0.438, p = 0.032), was disrupted in TRS + LRS (Fig. 4E). Altogether, these data suggest that the glutamate transporter EAAT3, appearing to be a key limiting factor in RESP, became dysregulated in TRS + LRS group.

### Association between NMDAR co-agonists and cognition

**Fig. 5:**
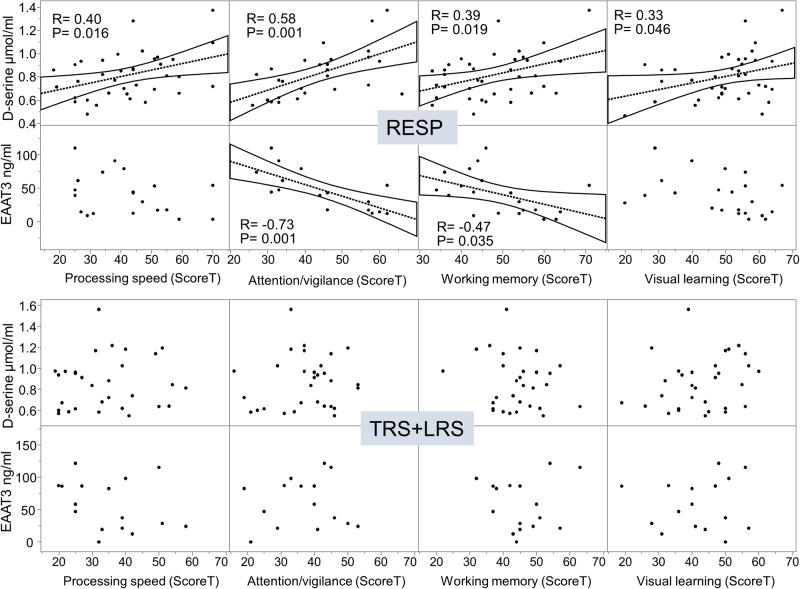
Association between plasma biomarkers and cognition only in RESP. Higher D-serine levels were associated with better processing speed (r = 0.40, p = 0.016), attention/vigilance (r = 0.58, p = 0.001), working memory (r = 0.39, p = 0.019) and visual learning (r = 0.33, p = 0.046) in RESP. In the TRS + LRS group, which displays significantly lower cognitive scores in all the cited domains, this association is absent. A negative correlation was found only in RESP between EAAT3 plasma levels and attention/vigilance (r = -0.73, p = 0.001) and working memory (r = -0.47, p = 0.035). No association between NMDAR co-agonist pathways and cognition was found in HC (Supplementary Fig. 3). All cognitive scores are based on age- and gender-standardized data (ScoreT).

Testing whether the observed dysregulation of the D-serine/glutamate pathways could underlie the worse cognitive deficit in the TRS+LRS compared to RESP subgroups, we investigated the relationship between the plasmatic levels of NMDAR co-agonist pathways biomarkers and cognitive scores in domains where TRS scored lower than RESP. Higher D-serine levels were associated with better processing speed (r = 0.396; p = 0.016), attention/vigilance (r = 0.581; p = 0.001), working memory (r = 0.389; p = 0.019) and visual learning (r = 0.326; p = 0.046) only in RESP (Fig. 5A; Supplementary Fig. 3). A negative correlation was also found only in RESP between EAAT3 levels and attention/vigilance (r = -0.73; p = 0.001) and working memory (r = -0.50; p = 0.040) (Fig. 5B; Supplementary Fig. 3). All statistical differences were corrected for multiple comparisons (see Methods). The associations were disrupted in TRS + LRS, pointing towards a critical compensatory process involving the D-Serine and glutamate pathways in the RESP but not the TRS or LRS groups.

## Discussion

In a prospective well-defined treatment resistant, FEP cohort, TRS and LRS patients showed worse cognitive and functioning impairments than RESP, with LRS as an intermediary phenotype between RESP and TRS. In the TRS + LRS group, our results uniquely highlighted a dysregulation of the D-serine pathway, with lower SRR and SHMT1 protein levels than in RESP group. We also revealed higher levels of the glutamate-cysteine transporter EAAT3 in TRS + LRS than in RESP, thus providing a novel molecular target for potential future clinical impact. Indeed, association studies between the sequential enzymes SRR and SHMT1 as well as between EAAT3 and glutamate indicate the potential involvement of SRR, SHMT and EAAT3 as critical limiting factors in individuals with treatment resistance. Also, an association between a better cognition with higher D-serine and lower EAAT3 levels only in RESP but disrupted in TRS + LRS group provide novel molecular insights into NMDAR hypofunction as possible underlying mechanisms leading to early cognitive deficits in individuals with treatment resistance.

To the best of our knowledge, the studied cohort, consisting of 697 individuals with 293 classified as patients, is one of the largest prospective monocentric cohorts for treatment resistant FEP to date. Our findings on cognitive impairment in this population represent a significant and comprehensive addition to the still limited literature. Moreover, this study marks the first analysis of cognition and biomarkers in FEP patients with treatment resistance.

Patients were assigned to TRS, LRS and RESP groups by applying the TRRIP criteria [3] through an automated selection process over 7 prospective timepoints. The utilization of systematic and prospective criteria for assessing response and resistance to antipsychotic treatment is noteworthy. It is to emphasize that this study stands out as one of the few where compliance confirmation was achieved through the quantification of plasma antipsychotic levels, enabling a meticulous categorization of patients. Of note, out of the 414 patients who were not assigned to any group, 248 constituted a continuous cohort and were excluded because of lack of data on treatment (collection of data on medication started in 2010); the remaining 166 could not be classified due to insufficient compliance, lack of clinical data (e.g. linked to drop out of the TIPP program) or very recent inclusion in the TIPP cohort. However, there were no significant differences between included and excluded patients (Supplementary Table 1). Additionally, the subgroup of patients participating to neurobiological research have been shown to be representative of the whole clinical cohort [53].

TRS and LRS patients displayed early cognition deficits and long-term functioning impairment compared to RESP. Compared to RESP group, TRS patients performed lower in 5 out of 6 neurocognitive MATRICS domains while LRS presented an intermediary phenotype, with lower scores only in visual and verbal learning. Our results, in line with previous findings from two smaller cohorts [4, 54], strongly suggest that cognitive deficits constitute an early feature in TRS and LRS. A major reason for the impact of cognitive deficits on people with psychosis is their association with functional outcomes [55, 56]. Given the prevalence of cognitive deficits in TRS and LRS patients, this relationship may explain lower social functioning in these groups compared to RESP.

Interestingly, although LRS patients showed resistance to only one drug over a 3-year follow-up, their GAF score never reached the threshold of 61, with a GAF profile more analogous to that of TRS than RESP (Fig. 2A). Our results revealed that individuals with LRS constitute an understudied group that may benefit from early detection and pro-active early intervention such as cognitive remediation and, as previously suggested [9], be candidates for early clozapine prescription.

Our findings highlight for the first time a dysregulation of the D-serine pathway in TRS + LRS patients. Indeed, the increase in SHMT1 and SRR levels in RESP compared to TRS + LRS may reflect a better response to antipsychotics. Similarly, a positive association between the two sequential enzymes SRR and SHMT1 was observed only in RESP, while this correlation was disrupted TRS + LRS group. This points to the involvement of compensatory mechanisms through the D-serine pathway in RESP group. Moreover, a positive correlation between the levels of substrates for each enzyme (reflecting the activity of each enzyme) was present only in TRS + LRS patients, indicating that SRR and SHMT1 enzymatic activities could represent key limiting factors in TRS + LRS. The latter could include deficient enzyme activities, SRR being a risk gene for schizophrenia [22–24, 26] and/or the limited availability of the substrates L-serine and/or D-serine (Fig. 3A). Consistently, a D-serine pathway modulation mediating increases of glycine/L-serine and D-serine/L-serine ratio may underlie the beneficial effects of clozapine treatment in TRS [32]. The association between a better cognition with higher D-serine levels only in RESP, but disrupted in TRS + LRS subgroups, underscores the ability of RESP to compensate by modulating the D-serine pathway, whereas TRS + LRS patients, as discussed above, may show limitations in SRR and SHMT1 activity.

Moreover, we highlighted a difference in glutamate pathway biomarkers between TRS + LRS and RESP, in line with previous evidence [10, 11, 13, 16]. Following the intriguing observation of an association between low brain glutamate with high blood glutamate levels only in patients, combined with a higher plasma glutamate level in TRS + LRS group, we revealed that plasma EAAT3 levels were abnormally higher in TRS + LRS than in RESP (Fig. 4D, right panel). The negative association between EAAT3 and glutamate found only in RESP suggests that EAAT3, potentially a key limiting factor in RESP, became dysregulated in TRS + LRS group. Interestingly, EAAT3 striatal transcript expression is decreased in schizophrenia [17] and SLC1A1, the gene encoding EAAT3, is a risk gene for schizophrenia [18]. Due to its predominant post-synaptic localization, EAAT3 can buffer nearby glutamate receptors and modulate glutamate neurotransmission and synaptic plasticity. As a co-transporter of cysteine, the rate-limiting substrate of glutathione synthesis, EAAT3 is also critically involved in the synthesis of intracellular glutathione and its ensuing protection from oxidative stress [20, 21, 57]. Converging clinical [48, 58–61] and preclinical [62–65] evidence supports the crucial role of the parvalbumin-GABAergic interneurons circuitry and their associated neuronal synchronization in cognitive processes. Due to their fast-spiking properties leading to high metabolic demand, parvalbumin interneurons are particularly vulnerable to oxidative stress [66, 67]. Combined with cumulative evidence of a reciprocal interaction between glutamatergic/NMDAR hypofunction and oxidative stress impacting on parvalbumin interneurons and cognition in schizophrenia [67–69], our finding of EAAT3 abnormalities as well as its association with cognition in RESP but disrupted in TRS + LRS group may provide a novel molecular target for improving cognition in individuals with treatment resistance.

Converging preclinical and clinical evidence support the critical involvement of NMDAR hypofunction in cognitive deficits [30, 70–73]. To our knowledge, this is the first time an NMDAR hypofunction mechanism-based biomarker has been associated with cognitive scores in FEP patients stratified by treatment resistance. Several studies have investigated the effect of antipsychotic treatment augmentation with NMDAR co-agonists (D-serine, D-cycloserine, glycine) [74, 75] but yielded mixed results [76]. Although long illness duration could have contributed to the mixed results, a lack of mechanism-based stratification may have played a pivotal role. If replicated, our findings would lay the groundwork for clinical trials aimed at testing cognitive improvement in patients stratified using mechanism-based biomarkers. This approach would entail targeting NMDAR through the utilization of existing NMDAR co-agonists [74, 75] or enhancing NMDAR activation by modulating co-agonist homeostasis and transport [76–78]. For instance, targeting the glycine transporter (GlyT-1) has been demonstrated to inhibit the uptake of the co-agonist glycine, thereby enhancing NMDAR activation [38, 79]. Notably, GlyT-1 inhibitors have exhibited the ability to reverse ketamine-induced working memory deficits in healthy individuals [80].

Although this is the largest monocentric cohort based on the TRRIP guidelines comparing FEP TRS patients to RESP published thus far, replication in a multicentric study would be needed. Due to the highly demanding nature of magnetic resonance spectroscopy alongside clinical, cognitive, and biofluid assessments, only a restricted number of individuals with treatment resistance could be examined.

Our results primarily rely on comprehensive blood measures of the glutamate and D-serine systems. An inherent limitation of using plasma biomarkers is the open question regarding their reflection of brain levels, notwithstanding our identification of a negative correlation between blood glutamate and brain glutamate in our patient cohort. Limited evidence is available regarding the significance of peripheral levels of NMDAR co-agonists, primarily due to the lack of direct access to brain levels; however, accumulating evidence suggests that peripheral D-serine levels are indicative of brain levels [81]. SRR is primarily expressed in the brain, although peripheral expression has also been detected in myocardial and kidney tubular cells [82, 83]. SHMT1 is expressed in most peripheral tissues and in the brain [82]. Therefore, measurement of protein levels in peripheral blood may be influenced by peripheral tissue expression. To overcome this limitation, our study not only focuses on absolute enzyme levels but primarily on the correlation between the substrate and product of the enzyme. This correlation serves as a proxy for enzymatic activity and is expected to be more relevant to function. The implications concerning neurobiological pathways should be further confirmed, possibly through a translational approach. Moreover, the role of other metabolites of the D-serine/glutamate pathways such as DAAO and 3-PDGH, which is involved in the L-serine production [23], was not analyzed and therefore could not be excluded.

Early stage psychosis represents a highly heterogeneous phenotype regarding clinical presentation, treatment response and illness course, ranging from transient psychotic experiences to treatment resistant schizophrenia [84]. Accordingly, the identification of biomarkers for the prediction of clinical trajectories and stratification of illness subtypes is critical for improving clinical, cognitive and functional outcomes. Our study showed early and persistent poor social functioning in TRS and LRS, possibly linked to significant cognition deficits in these groups already present in the early phases, compared to RESP. Our findings in LRS patients, although needing replication, prompt new insights into the clinical importance of a single episode of treatment resistance and challenge the adequacy of existing clinical guidelines concerning this patient category. Our results also showed the NMDAR co-agonist pathways are potential underlying molecular mechanisms of treatment resistance, with D-serine and EAAT3 being associated with cognitive performance in RESP and possible compensating mechanisms in this group. Further research and replication of these results is of paramount importance to open ways to new treatment approaches based on mechanisms to improve cognition and functioning in individuals with treatment resistance.

## Supplementary information

Supplementary information

Supplementary table 1

Supplementary figure 1

Supplementary figure 2

Supplementary figure 3
