# Supplementary material for: Neurocognition and NMDAR co-agonists pathways in individuals with treatment resistant first-episode psychosis: a 3-year follow-up longitudinal study
Source: Mol Psychiatry. 2024 Jun 7;29(11):3669–79. doi: 10.1038/s41380-024-02631-4 (PMC11541217; doi:10.1038/s41380-024-02631-4)
Supplement: Supplementary file 1 — Supplementary information [file 41380_2024_2631_MOESM1_ESM.docx]

**Supplementary Information: Supplementary Table and Supplementary Figures legends**

**Supplementary Table 1. Missing data: comparing the observed characteristics between patients who were included in the study** (i.e patients with complete data on treatment compliance and antipsychotic dosage, N= 293) **and patients who were excluded** (i.e patients with incomplete data on compliance and/or antipsychotic dosage, N= 414). There was no difference in age at psychosis onset, age at baseline, BMI, disorder duration (i.e. time from psychosis onset to entry in the TIPP program), or gender. There was no difference in age at psychosis onset, gender, BMI, disorder duration between the patients included in the analysis and excluded patients.

**Supplementary Figure 1. Plasma levels of key markers of the D-serine and glutamate pathways were assessed in HC, RESP, LRS and TRS separately.** TRS and LRS groups showed no difference in levels of SHMT1, SRR, D-serine, L-serine and glutamate.

**Supplementary Figure 2.** **mRNA levels abnormalities of the glutamate and cysteine transporter EAAT3 in patients compared to HC.** Among various glutamate transporters analyzed in skin-derived fibroblasts of individuals with long-term schizophrenia, preliminary results showed mRNA levels abnormalities of the glutamate and cysteine transporter EAAT3 compared to HC (mean difference 0.4058, [95%CI, 0.0056-0.8061]; p=0.04).

**Supplementary Figure 3.** **Correlation color map showing the association between cognition and glutamatergic biomarkers plasma levels.** No correlation was found between plasma glutamate levels and cognitive scores. Associations between D-Serine and EAAT3 levels and cognition are detailed in figure 5.
