# Supplementary material for: Neurocognition and NMDAR co-agonists pathways in individuals with treatment resistant first-episode psychosis: a 3-year follow-up longitudinal study
Source: Mol Psychiatry. 2024 Jun 7;29(11):3669–79. doi: 10.1038/s41380-024-02631-4 (PMC11541217; doi:10.1038/s41380-024-02631-4)
Supplement: Supplementary file 2 — Supplementary table 1 [file 41380_2024_2631_MOESM2_ESM.pdf]

Supplementary Table 1

Supplementary Table 1. Missing data				
	Patients		Incl. vs Excl.	
**Variable**	**Included (293)**	**Excluded (414)**	**P-value**	**Cohen's d (df)**
Age at psychosis onset, mean (SD), y	23.3 (5.0)	22.9 (5.2)	0.331	0.079 (610)
Age at baseline, mean (SD), y	24.8 (5.0)	24.2 (4.6)	0.205	0.126 (404)
Body mass index, mean (SD)	24.4 (4.2)	23.7 (3.8)	0.429	0.166 (109)
Disorder duration, mean (SD), days	121 (264)	154 (354)	0.515	-0.107 (116)
Gender				
Male, No (%)	72%	66%		
Female, No (%)	28%	34%	0.19	χ2 (3.23)
Ethnicity, no.	75 C, 17 A, 13 M, 188 O	104 C, 17 A, 20 M, 273 O	NA	NA
Diagnosis no.				
Schizophrenia	183	272	NA	NA
Schizophreniform disorder	28	38	NA	NA
Schizoaffective disorder	29	43	NA	NA
Major depression w/ psychotic feat.	13	18	NA	NA
Bipolar disorder	14	21	NA	NA
Other	26	22	NA	NA
Years of education, mean (SD)				
Patient	12.3 (2.7)	13.7 (3.1)	**0.017**	-0.480(118)
GYOE patient	1.84 (0.65)	2.1 (0.65)	**0.043**	-0.405 (118)
Mother	11.2 (4.4)	12.3 (4.9)	0.301	-0.252 (82)
GYOE mother	1.75 (0.73)	1.8 (0.78)	0.491	-0.167 (82)
Father	12.1 (5.1)	12.5 (4.3)	0.705	-0.092 (79)
GYOE father	1.88 (0.83)	2.00 (0.718)	0.536	-0.151 (79)

Footnotes: [a]degree of freedom; [b]standard deviation; [c]years; [d]number; [e]ethnicity: C, Caucasian; A, African; M, mixed; O, other; [f]other includes schizotypal personality disorder, autism spectrum disorder, brief psychotic disorder, psychotic disorder not otherwise specified; [g]grade of attained education.
