# Supplementary figures and images for: Neurocognition and NMDAR co-agonists pathways in individuals with treatment resistant first-episode psychosis: a 3-year follow-up longitudinal study
Source: Mol Psychiatry. 2024 Jun 7;29(11):3669–79. doi: 10.1038/s41380-024-02631-4 (PMC11541217; doi:10.1038/s41380-024-02631-4)

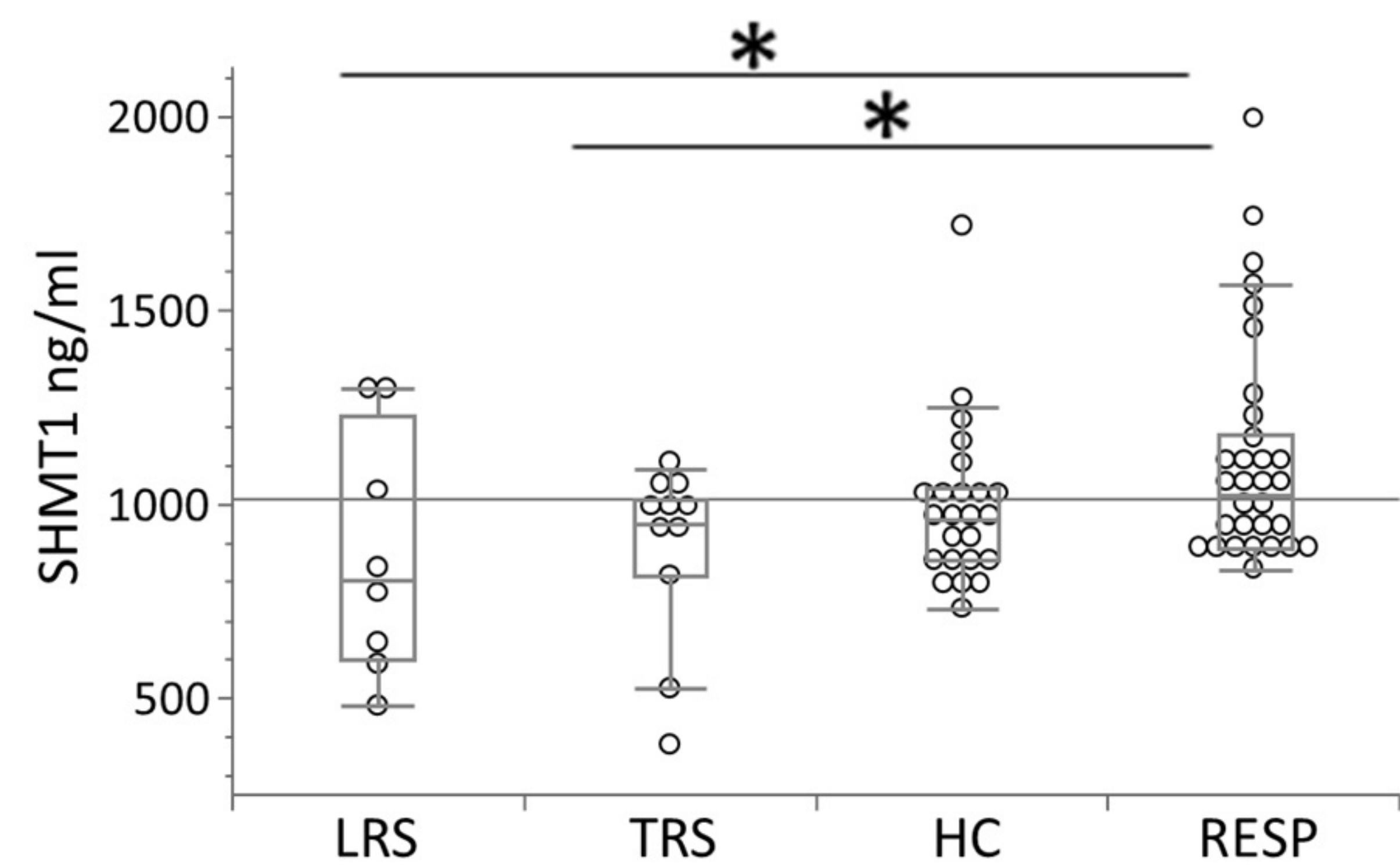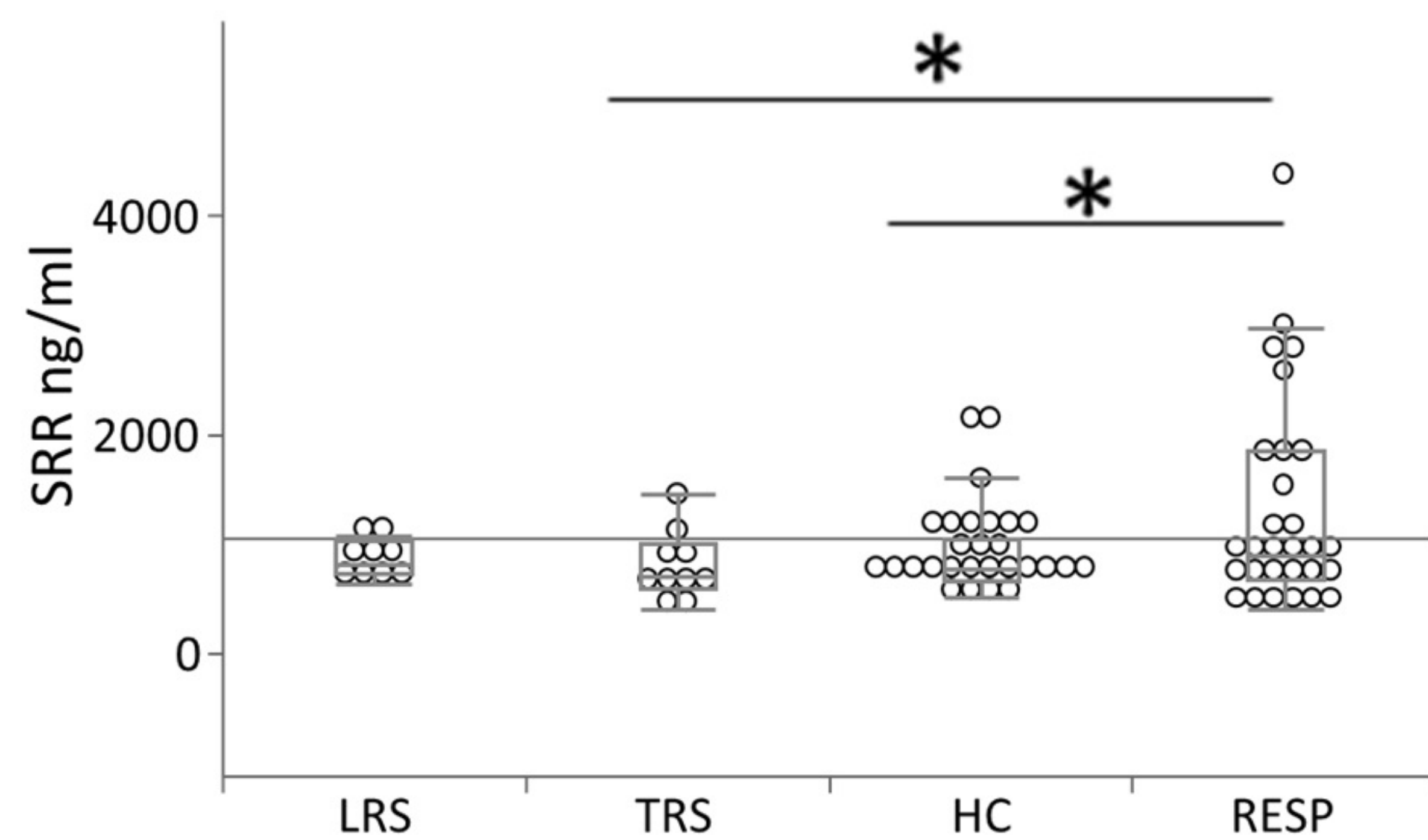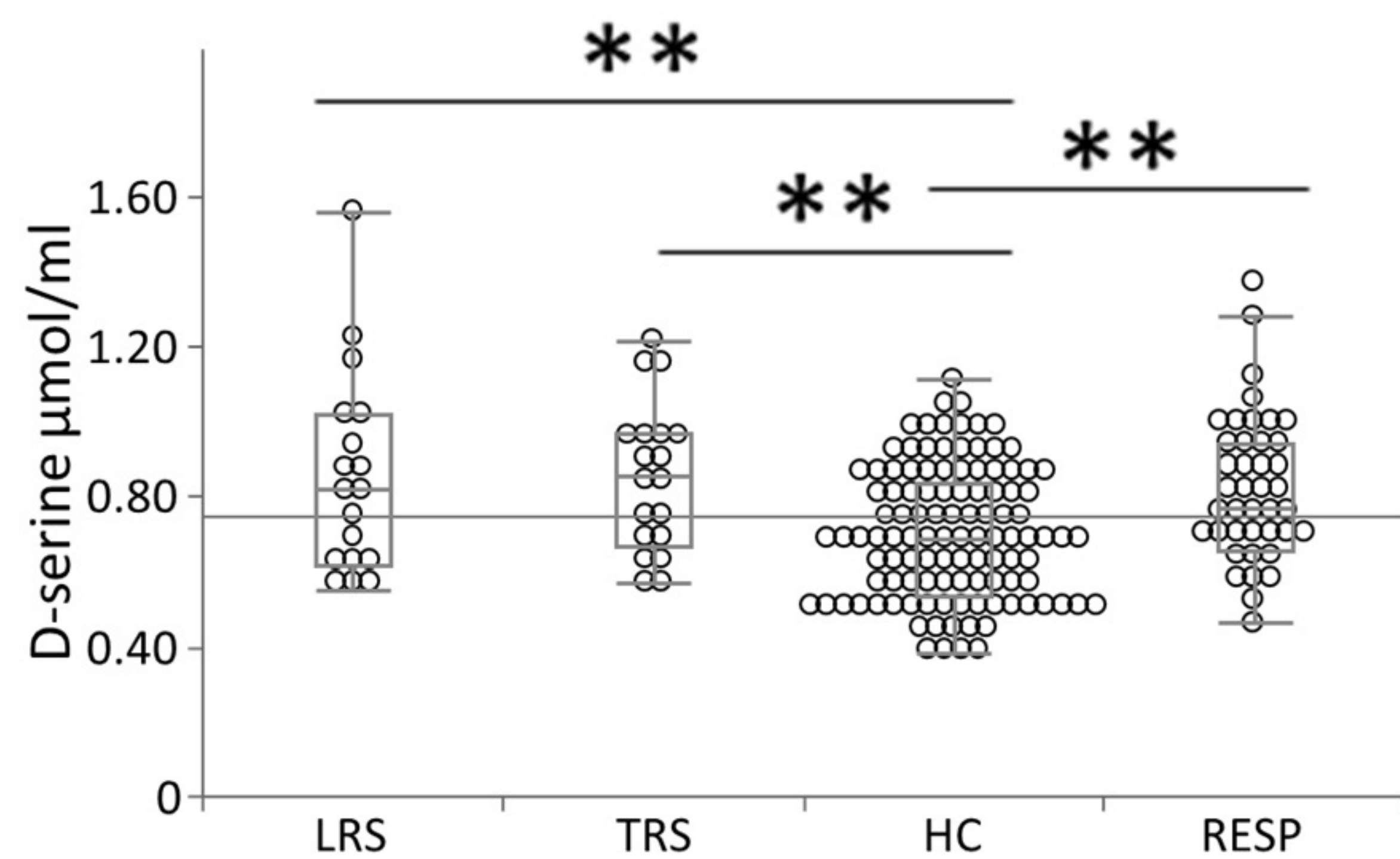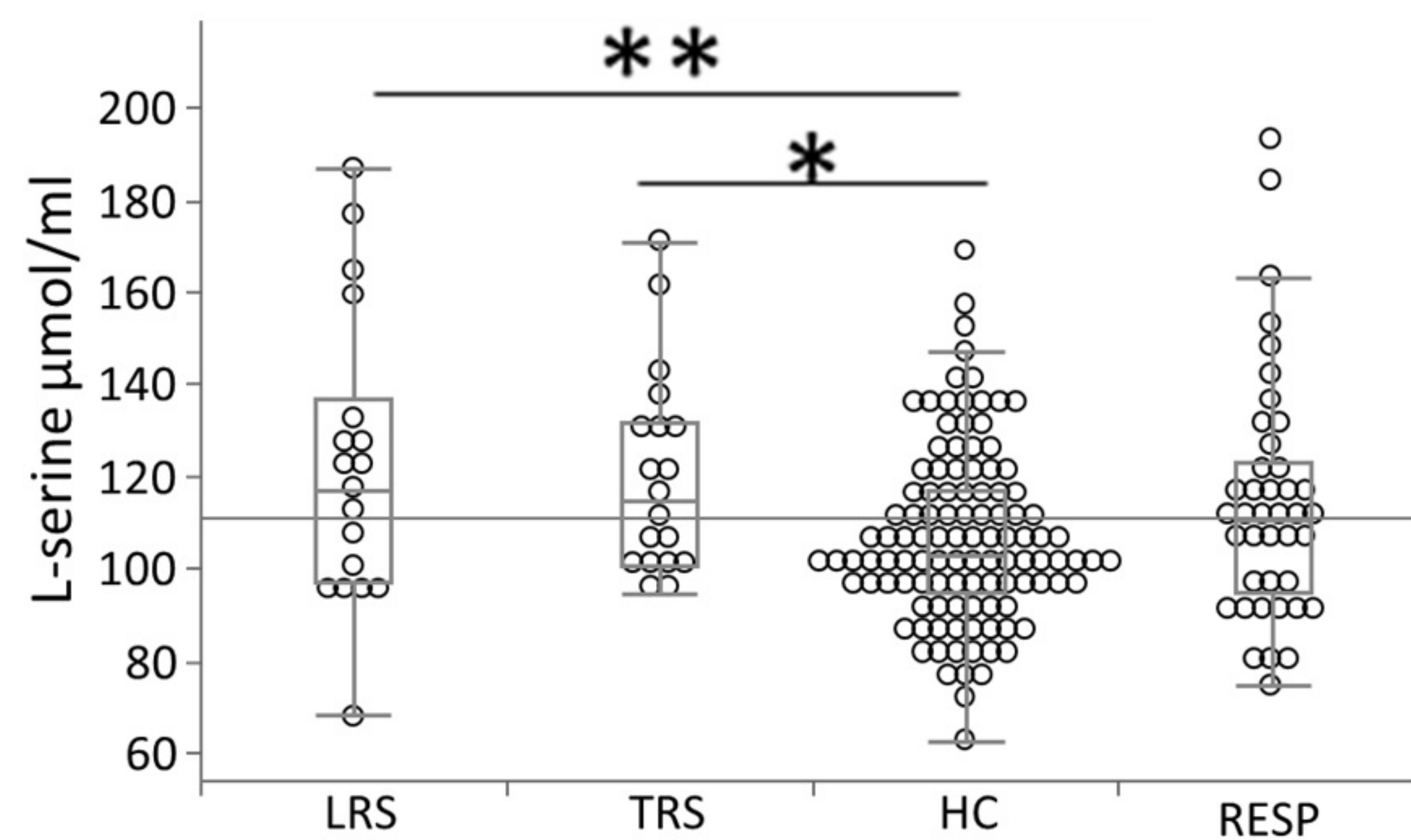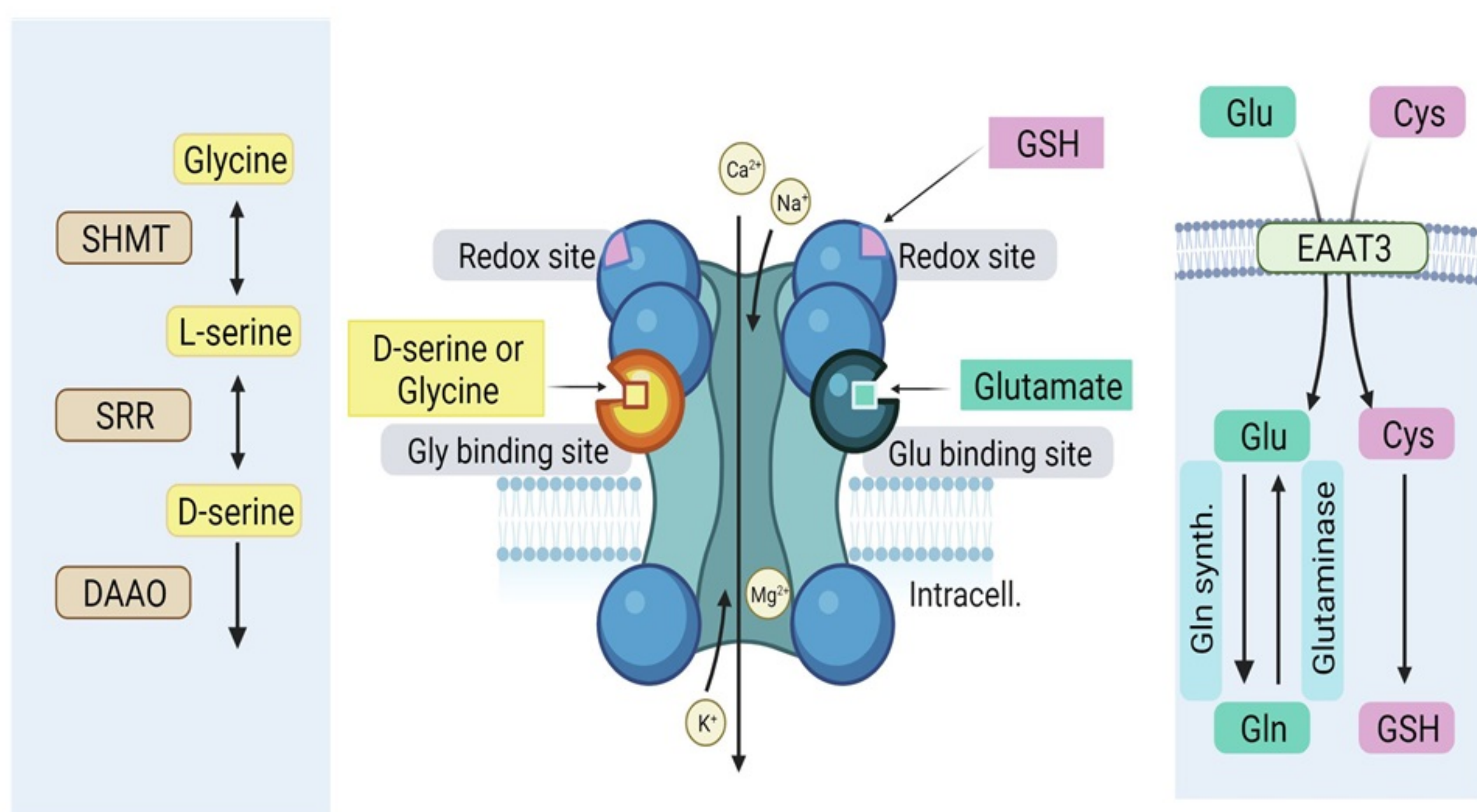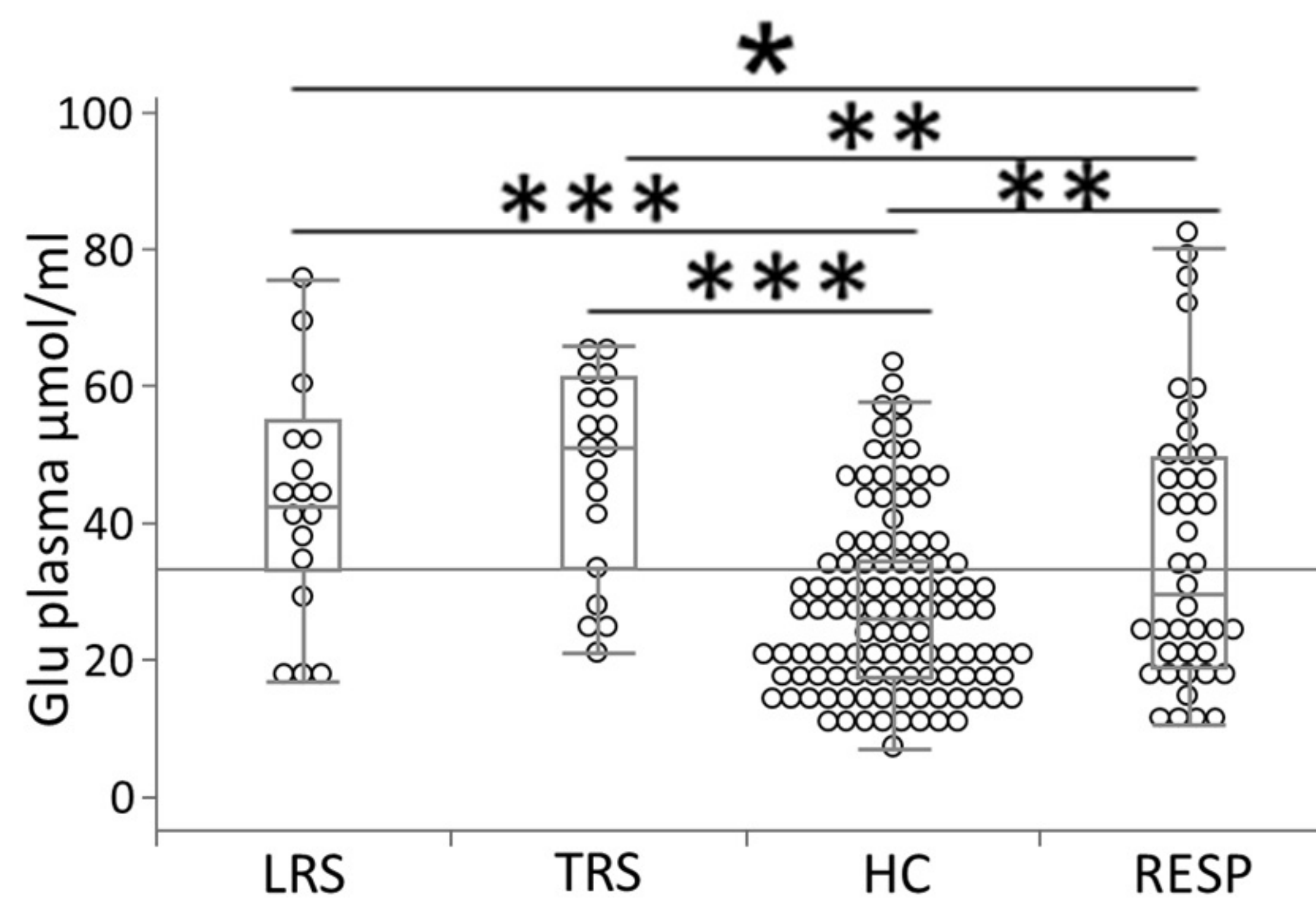

Supplement: Supplementary file 3 — Supplementary figure 1 [file 41380_2024_2631_MOESM3_ESM.pdf]

EAAT3 mRNA plasma

8  
6  
4  
2  
0

HC

Patients

\*

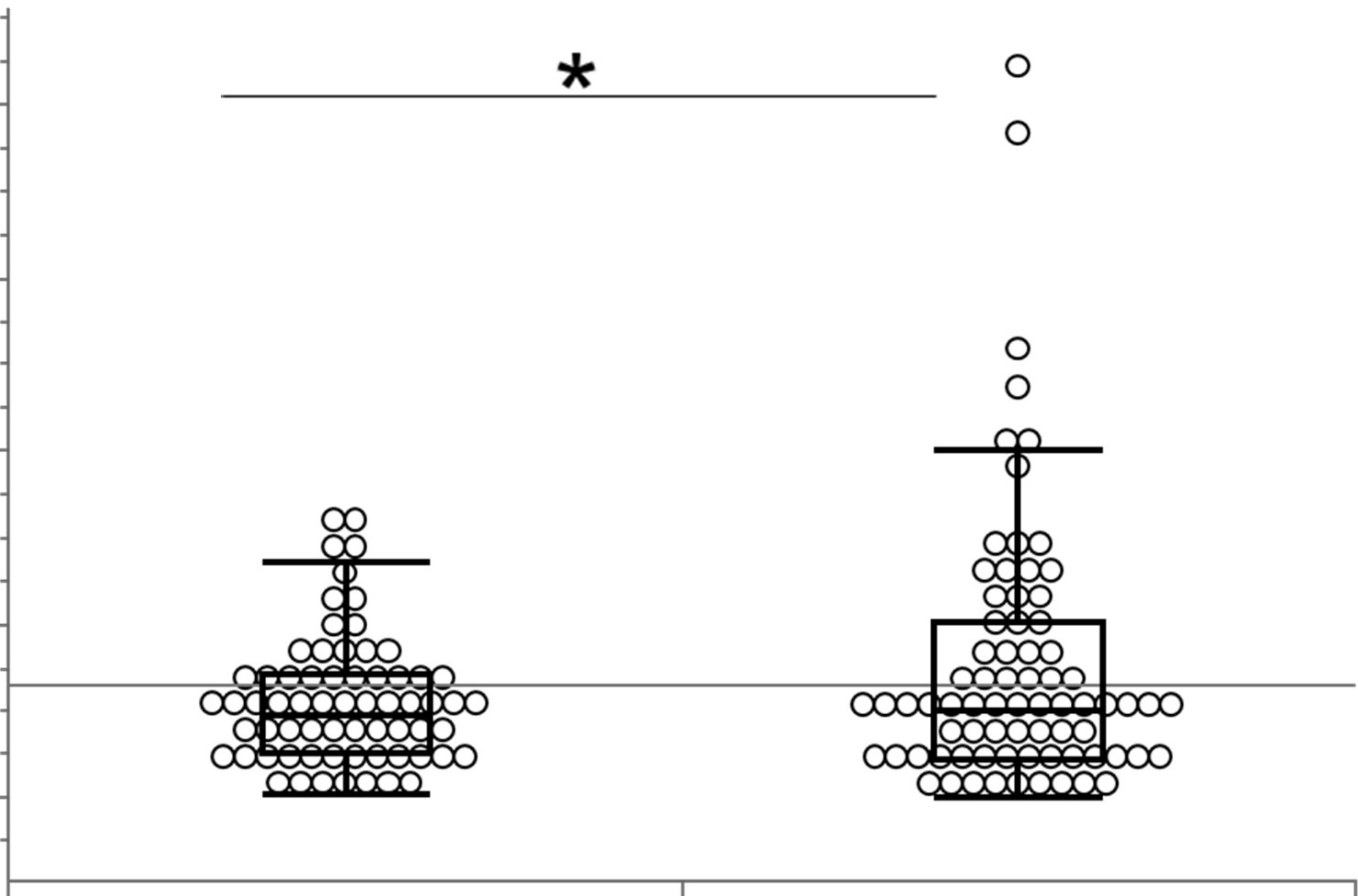

Supplement: Supplementary file 4 — Supplementary figure 2 [file 41380_2024_2631_MOESM4_ESM.pdf]
