# Supplementary material for: Neurocognition and NMDAR co-agonists pathways in individuals with treatment resistant first-episode psychosis: a 3-year follow-up longitudinal study
Source: Mol Psychiatry. 2024 Jun 7;29(11):3669–79. doi: 10.1038/s41380-024-02631-4 (PMC11541217; doi:10.1038/s41380-024-02631-4)

		HC					RESP					TRS+LRS				
		R	N	Lower 95%	Upper 95%	P value	R	N	Lower 95%	Upper 95%	P value	R	N	Lower 95%	Upper 95%	P value
**Processing speed (ScoreT)**	**D-Ser**	0.04	96	-0.164	0.237	0.712	0.40	36	0.078	0.642	0.0166*	0.10	31	-0.268	0.435	0.610
**Processing speed (ScoreT)**	**Glu**	0.13	96	-0.075	0.320	0.217	-0.09	36	-0.404	0.249	0.613	0.20	31	-0.166	0.518	0.280
**Processing speed (ScoreT)**	**EAAT3**	-0.30	9	-0.804	0.455	0.433	-0.33	20	-0.674	0.132	0.155	-0.31	16	-0.701	0.215	0.236
**Attention/vigilance (ScoreT)**	**D-Ser**	-0.14	93	-0.333	0.067	0.184	0.58	27	0.258	0.787	0.0015*	0.11	30	-0.264	0.449	0.577
**Attention/vigilance (ScoreT)**	**Glu**	0.12	93	-0.087	0.315	0.256	0.03	27	-0.355	0.405	0.884	0.10	30	-0.270	0.444	0.600
**Attention/vigilance (ScoreT)**	**EAAT3**	-0.17	7	-0.819	0.667	0.710	-0.73	16	-0.902	-0.376	0.0012*	0.04	15	-0.483	0.541	0.889
**Working memory (ScoreT)**	**D-Ser**	-0.08	99	-0.269	0.124	0.458	0.39	36	0.069	0.636	0.0192*	-0.16	30	-0.492	0.212	0.398
**Working memory (ScoreT)**	**Glu**	0.04	99	-0.157	0.237	0.684	0.09	36	-0.244	0.408	0.594	-0.24	30	-0.551	0.134	0.205
**Working memory (ScoreT)**	**EAAT3**	0.22	9	-0.517	0.773	0.561	-0.47	20	-0.757	-0.038	0.0353*	0.03	16	-0.474	0.517	0.916
**Verbal learning (ScoreT)**	**D-Ser**	-0.10	99	-0.296	0.095	0.302	0.31	37	-0.013	0.578	0.060	0.28	30	-0.094	0.579	0.140
**Verbal learning (ScoreT)**	**Glu**	0.04	99	-0.157	0.237	0.680	-0.14	37	-0.442	0.196	0.419	-0.17	30	-0.498	0.205	0.377
**Verbal learning (ScoreT)**	**EAAT3**	-0.36	9	-0.827	0.400	0.341	-0.38	21	-0.695	0.067	0.093	-0.05	15	-0.548	0.475	0.862
**Visual learning (ScoreT)**	**D-Ser**	-0.02	99	-0.214	0.181	0.865	0.33	38	0.006	0.584	0.0465*	0.23	31	-0.135	0.540	0.213
**Visual learning (ScoreT)**	**Glu**	0.02	99	-0.183	0.212	0.883	0.04	38	-0.282	0.357	0.804	-0.03	31	-0.376	0.332	0.892
**Visual learning (ScoreT)**	**EAAT3**	0.24	9	-0.506	0.779	0.537	-0.40	22	-0.704	0.024	0.064	0.08	16	-0.435	0.552	0.774

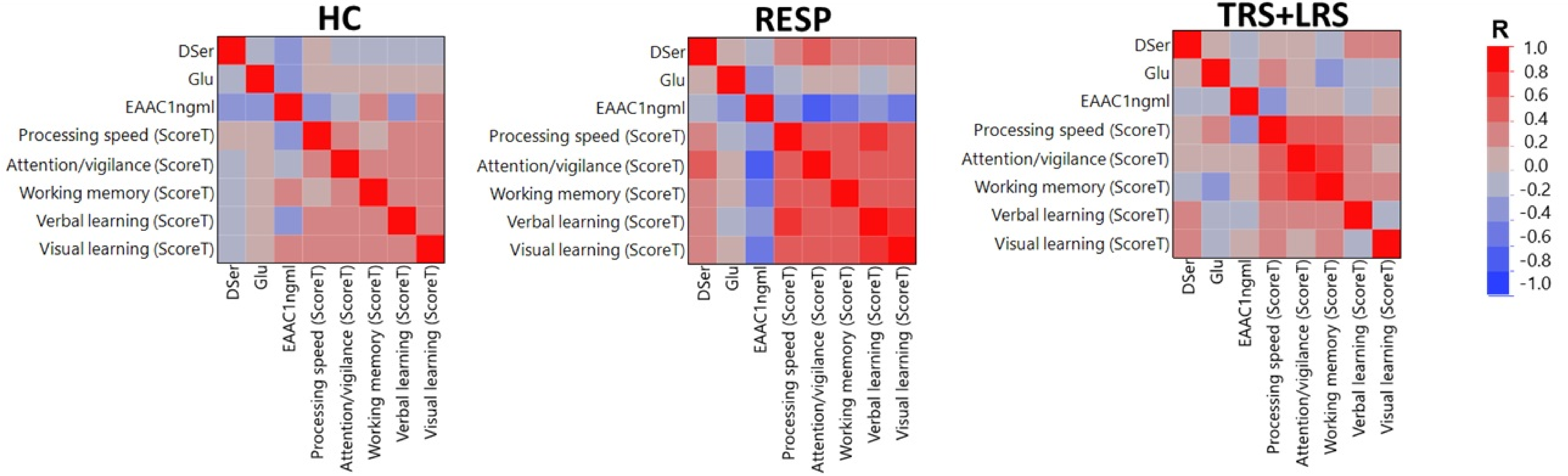

Supplement: Supplementary file 5 — Supplementary figure 3 [file 41380_2024_2631_MOESM5_ESM.pdf]
